# Latest updates on pathogenesis mechanisms and management strategies for cytokine release syndrome, neurotoxicity, and hemophagocytic lymphohistiocytosis related to CAR-T cell therapies

**DOI:** 10.1007/s00277-025-06467-y

**Published:** 2025-06-19

**Authors:** Xin Wang, Xiaoyuan He, Tao Zhang, Jile Liu, Mingfeng Zhao

**Affiliations:** 1 First Center Clinical College, Tianjin Medical University, Tianjin, 300070 China; 2 Department of Hematology, Tianjin Thrombosis and Hemostasis Institute, Tianjin First Central Hospital, No.2 Baoshan West Road, Xiqing District, Tianjin, 300192 China; 3 Nankai University School of Medicine, Nankai University, Tianjin, 300071 China

**Keywords:** Chimeric antigen receptor (CAR)-T cell therapies, Cytokine release syndrome, Immune effector cell-associated neurotoxicity syndrome, Hemophagocytic lymphohistiocytosis

## Abstract

Nowadays, chimeric antigen receptor (CAR) -T cell therapy has shown significant efficacy in treating hematological tumors, with an obvious increase in patient survival rates. However, with the widespread application of CAR-T, the incidence of CAR-T related adverse events has gradually increased, including cytokine release syndrome (CRS), immune effector cell associated neurotoxicity syndrome (ICANS), and hemophagocytic lymphohistiocytosis (HLH). These complications may be life-threatening, so early diagnosis and intervention treatment are crucial for the prognosis of patients. In this review, we first comprehensively summarize the latest insights into the pathogenesis and clinical manifestations of CRS, ICANS, and HLH after CAR-T, with a focus on elaborating on the specific mechanisms and related pathways of the inflammatory storm caused by a large number of cytokines after CAR-T. We also discussed the established prevention and management strategies for the three complications mentioned above, and demonstrated the effectiveness of the treatment by introducing the therapeutic effects of various treatment methods in current clinical or preclinical trials. In addition, we provide a prospective perspective on the measures and modifications currently being studied to mitigate the toxicity associated with CAR-T cell therapy.

## Introduction

Chimeric antigen receptor (CAR)-T cell therapies, with their precise targeting of tumor cells, have achieved notable success in treating malignant hematological diseases, including acute lymphocytic leukemia (ALL) [1], non-Hodgkin lymphoma (NHL) [2], and multiple myeloma (MM) [3]. Despite these successes, CAR-T cell therapies are also associated with adverse events such as cytokine release syndrome (CRS), immune effector cell-associated neurotoxicity syndrome (ICANS), and hemophagocytic lymphohistiocytosis (HLH), which pose complex challenges in clinical management. A meta-analysis reported an incidence of CRS related to CAR-T treatment of approximately 55.3%, with severe CRS occurring in about 18.5% of cases [4]. Published data revealed that nearly 46% of B-ALL patients [5], 13% of B-cell lymphoma patients [6] treated with anti-CD19 CAR-T treatment, and 41% of MM patients treated with BCMA CAR-T [7] experience severe CRS (grade ≥ 3). ICANS, the second most common toxicity associated with CAR-T cell therapy, has an incidence ranging from 2 to 64% for all levels and 0–50% for severe cases [8]. Recently, the attention given to CAR-T cell-associated HLH has grown due to its high morbidity and mortality. In two Phase I clinical trials, 32.7% and 35.6% of patients respectively developed HLH [9, 10], and the mortality rate for HLH can reach up to 80% [11–13].

CRS, ICANS, and HLH share the commonality of being consequences of CAR-T cell activation in response to tumor recognition, leading to an excessive release of cytokines and damage to multiple organ systems. However, there are several differences in their pathogenesis and management strategies. Tocilizumab is currently the first-line pharmacotherapy for CRS according to the American Society for Transplantation and Cellular Therapy (ASTCT) guidelines [14], while corticosteroids are used for severe or tocilizumab-refractory CRS [15]. Tocilizumab is not recommended for ICANS due to its potential to elevate IL-6 levels in the cerebrospinal fluid (CSF), potentially worsening ICANS [6] Although tocilizumab can significantly alleviate CRS, patients may still progress to CAR-T cell-related HLH. This suggests that other factors may play a more dominant role in the pathogenesis of HLH than IL-6 [16]. Given the high incidence and mortality rates associated with severe CRS, ICANS, and HLH, understanding their pathogenesis mechanisms is essential for optimizing management. This review aims to provide the latest updates on the pathogenesis mechanisms and management strategies for CRS, ICANS, and HLH related to CAR-T cell therapies.

## Pathogenesis mechanisms of CRS related to CAR-T cell therapies

Cytokines, a class of soluble regulatory proteins or glycoproteins, are synthesized and secreted by immune cells such as CAR-T cells, T cells, macrophages, dendritic cells (DCs), and non-immune cells including tumor cells, endothelial cells, and fibroblasts upon stimulation. They play a crucial role in regulating cellular processes like growth, differentiation, tissue development, repair, and immune responses [17]. CRS, a systemic inflammatory response, commonly complicates immunotherapy for hematological tumors, particularly following CAR-T cell therapies, bispecific antibodies, and immune checkpoint inhibitors. The median onset of CRS is typically within the first week post CAR-T cell administration [18]. Clinical studies have observed that high tumor load is an important clinical factor related to the incidence rate and severity of CRS, along with other risk factors such as tumor type, CAR-T cell infusion dose, and peak CAR-T cell expansion [15, 19]. Over-activation of immune effector cells leads to the release of a multitude of cytokines and causes systemic symptoms including fever, headache, hypotension, hypoxia, rash, and joint and muscle pain. In severe cases, CRS can progress to multi-organ dysfunction and life-threatening toxic reactions affecting the respiratory, circulatory, and nervous systems [20]. Thus, a comprehensive understanding of the pathogenesis of CRS related to CAR-T therapies is essential for improved management.

**Fig. 1:**
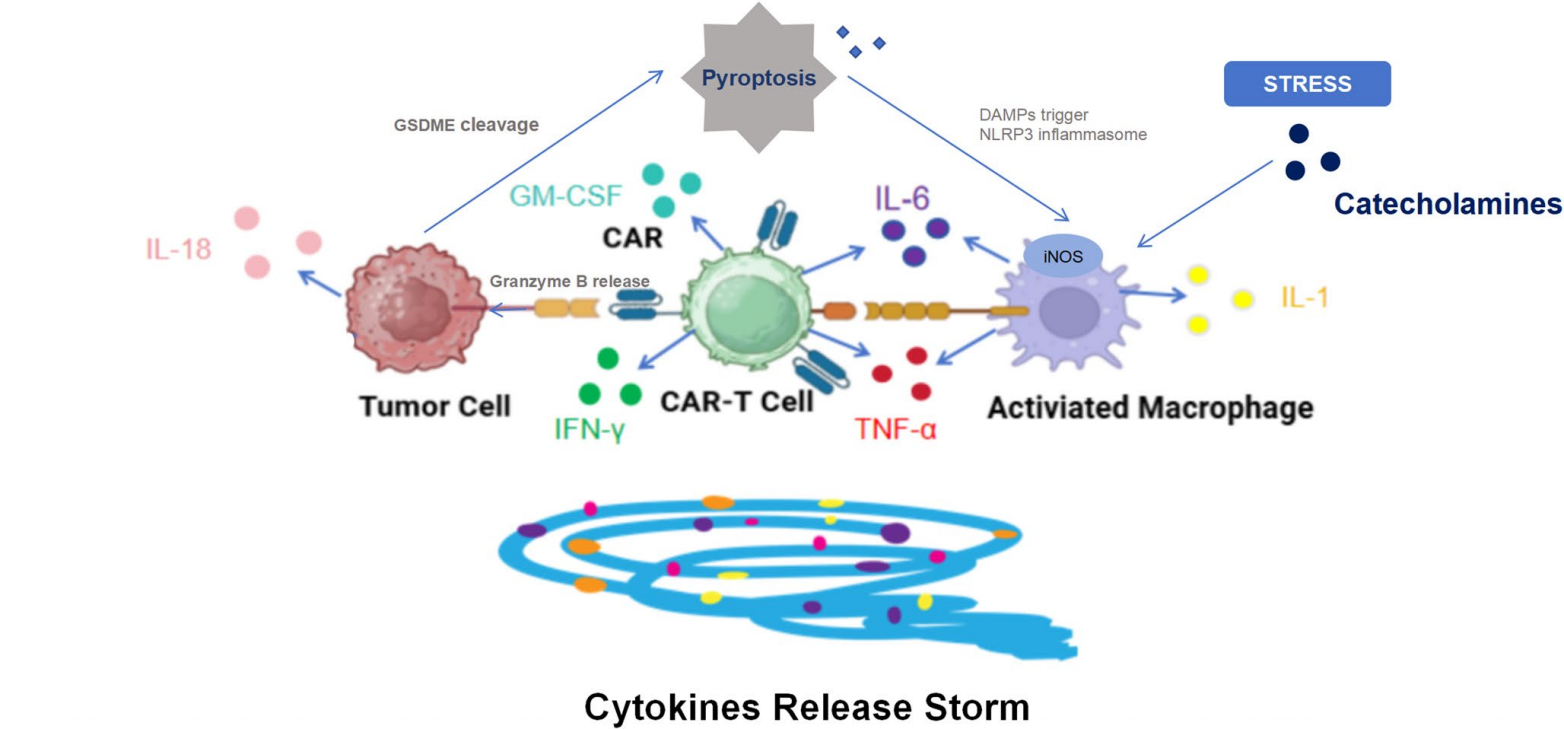
Schematic diagram of cytokines (such as IL-6, IL-1, GM-CSF) and immune cells (CAR-T cells, macrophages) involved in the pathogenesis of CRS. The blue arrows indicate the secretion of various cytokines and catecholamines, as well as the pyroptosis process of target tumor cells, which collectively contribute to the occurrence and development of CRS

The pathogenesis of CRS can be divided into three stages [15]: Initially, CAR-T cells are transported to the tumor site after infusion and recognize antigen positive target cells. Subsequently, CAR-T cells proliferate in the tumor micro-environment and produce cytokines, while endogenous immune cells participate in tumor cell lysis. Finally, the elevated levels of cytokines in peripheral blood can lead to the expansion of CAR-T cell populations, triggering systemic inflammatory responses and resulting in CRS. Therefore, CRS is not merely a result of interactions between tumor and CAR-T cells but also involves interactions with other immune and non-immune cells, as well as a plethora of cytokines and chemokines [21]. The key pathogenesis mechanism of CRS is shown in Fig. 1.

### Role of IL-6 in CRS

Interleukin-6 (IL-6), a secreted protein, plays a pivotal role in acute and chronic inflammation by regulating the differentiation, proliferation, migration, and apoptosis of target cells [22]. It is involved in B and T cell differentiation, bone homeostasis, immune responses, cancer development, and metabolic processes [23]. It is produced by various cells including monocytes, activated T cells, vascular endothelial cells, and fibroblasts [24]. Numerous studies have confirmed that IL-6 plays an important role in CRS and its level is correlated with the severity of CRS [25]. IL-6 activates downstream signaling pathways such as JAK-STAT and MAPK-PI3K by binding to its receptor IL-6R, leading to the release of a large number of pro-inflammatory factors (such as TNF-α, IL-1β) and anti-inflammatory factors (such as IL-10), forming a cytokine storm. In addition, IL-6 can also conduct trans signaling through soluble IL-6R (sIL-6R), activate widely expressed gp130, and further amplify inflammatory signals [26]. Researchers have found that monocytes and macrophages are the primary sources of IL-6 during CRS [27, 28]. Therefore, we believe that this syndrome can be prevented and treated by monocyte depletion or blocking IL-6 receptor. Tocilizumab, an IL-6 receptor antagonist which can clear overloaded IL-6 and reduce inflammatory damage, has been approved by the Food and Drug Administration (FDA) for the treatment of CRS following CAR-T cell therapy. It has been demonstrated that the use of tocilizumab benefits most CRS patients and has good clinical efficacy in some retrospective data analysis [14, 29]. For example, clinical trial data showed that the efficacy of tocilizumab in severe CRS patients is over 70% [14].

### Role of IL-1 in CRS

The Interleukin-1 (IL-1) family, a class of multifunctional cytokines primarily consisting of IL-1α and IL-1β, activate a complex cascade of mediators, including cytokines, chemokines, and lipid mediators, playing a crucial role in initiating and coordinating inflammation and both innate and adaptive immunity [30, 31]. The following studies indicated that IL-1 plays an important role in CRS. The production of IL-1 is usually triggered by pathogen associated molecular patterns (PAMPs, such as bacterial lipopolysaccharides) or damage associated molecular patterns (DAMPs, such as ATP). NLRP3 inflammasome is a key platform for IL-1 maturation in CRS, immune cells such as macrophages and monocytes are overactivated, leading to a significant activation of NLRP3 inflammasomes and the release of a large amount of IL-1 [32]. The activation of IL-1 signaling is the core driving force behind the inflammatory response in CRS. IL-1 induces the production of a large number of inflammatory factors through the NF-κB and MAPK pathways, driving the inflammatory cascade reaction [31]. IL-1, plays a core role throughout the entire pathological process of CRS, from the release of early inflammatory factors to late stage multi organ dysfunction [33]. IL-1 not only induces the production of inflammatory factors such as IL-6 and TNF-α, forming a positive feedback loop and further amplifying the inflammatory response, which is the core characteristic of the pathological and physiological mechanism of CRS [25, 26]. IL-1 can also activate immune cells and intensify immune responses; activating endothelial cells leads to increased vascular permeability, causing tissue edema and hypotension [34–36], as well as inducing metabolic disorders. The severity of CRS increases with the production of IL-1 by activated macrophages and monocytes. Therefore, regulating macrophage function or blocking IL-1 signaling can reduce CRS-related mortality, highlighting the critical role of macrophages in CRS pathogenesis and suggesting that IL-1 is a potential target for alleviating the severity of CRS [33, 34, 37]. Multiple animal models and clinical trials have shown that blocking IL-1-induced signal transduction with the IL-1R antagonist anakinra protected mice from weight loss, fever, and CRS-related death [25]. Notably, the anti-tumor efficacy of CAR-T cells was maintained while inhibiting CRS when prophylactic IL-1 inhibition was initiated. This discovery provides a new approach for designing CAR constructs that secrete IL-1R antagonists (IL-1Ra), which may reduce CRS related mortality rates without compromising anti-tumor efficacy. IL-1β, the primary form of IL-1, is involved in various autoimmune inflammatory responses and cellular activities, including cell proliferation, differentiation, apoptosis, inflammation, autoimmunity, and tumor development [37–39]. The monoclonal antibody canakinumab against IL-1β showed therapeutic potential for treating CAR-T-related CRS in vitro [40]. In the treatment section below, an increasing number of studies have demonstrated the effectiveness of IL-1 receptor antagonists like anakinra and monoclonal antibodies like canakinumab against IL-1β in treating CRS and reducing CRS-related mortality.

### Role of IFN-γ in CRS

Interferon-gamma (IFN-γ), as a key pro-inflammatory soluble cytokine, plays an important role in the occurrence and development of CRS. IFN-γ is mainly produced by activated T cells (especially Th1 cells and cytotoxic T cells), macrophages, NK cells, and NKT cells. In CRS, the production of IFN-γ is typically activated by immune cells (such as CAR-T cells) recognizing target cells. IFN-γ can bind to the IFN-γ receptor (IFNGR) on the surface of target cells and activate the Janus kinase (JAK) and signal transducer and activator of transcription (STAT) pathways. This engagement is essential for immune regulation, cell proliferation, apoptosis, and anti-tumor activities [41, 42]. The specific mechanism of IFN-γ in CRS is reflected in its ability to activate macrophages and monocytes to release inflammatory factors, as well as induce endothelial cell activation and increase vascular permeability to promote inflammatory cell infiltration [43, 44]. Zhou Z’s team proposed that the release of IFN-γ may involve the granzyme A and B pathways, and deemed that the severity of CRS is positively correlated with granzyme levels [45]. Studies have highlighted the significant role of IFN-γ in the development of CRS, with potential strategies to mitigate cytokine storms by down-regulating IFN-γ using short hairpin RNA (shRNA) without compromising therapeutic efficacy. A novel shRNA targeting IFN-γ (shIFN-γ) integrated into the CD19CAR gene has been shown to maintain both potency and safety [46]. Given the crucial role of IFN-γ in CRS, clinical studies have demonstrated the efficacy of the IFN-γ monoclonal antibody emapalumab in controlling CAR-T induced CRS, which has been confirmed in relevant case reports [16, 47].

### Role of TNF-α in CRS

Tumor necrosis factor-alpha (TNF-α) is a pivotal cytokine that bridges inflammation and the immune system. TNF-α is secreted by macrophages through the activation of pattern recognition receptors such as toll-like receptors, T lymphocytes, neutrophils, and natural killer (NK) cells, as well as non-immune cells like endothelial cells and astrocytes [48, 49]. TNF-α exerts its effects by binding to two receptors, TNFR1 and TNFR2. TNFR1, equipped with a death domain in its cytoplasmic region, primarily mediates the activation of inflammation and programmed cell death associated with tissue damage, which is a key pathway in the development of CRS. After activation of TNFR1, the NF-κB and MAPK signaling pathways are activated and promote transcription of multiple pro-inflammatory genes. Subsequently, it produces pro-inflammatory effects similar to cytokines such as IL-6 and IFN-γ mentioned above [50, 51]. Wang J have demonstrated that patients with refractory/relapsed acute B lymphoblastic leukemia (B-ALL) exhibit a significant increase in TNF-α levels following CAR-T cell therapy, with peak serum levels correlating with CRS severity [52]. In a clinical study involving eight patients with refractory/relapsed multiple myeloma (R/R MM) who received BCMA CAR-T cell infusion, TNF-α levels significantly increased during CRS episodes. Treatment with etanercept, a TNF-α inhibitor, as monotherapy for CAR-T-induced CRS resulted in successful resolution in three patients, without any etanercept related adverse events. In vitro experiments further support that etanercept can mitigate CRS without affecting CAR-T cell proliferation or efficacy against MM cells [53]. The above findings suggest that the use of etanercept monotherapy for CRS post CAR-T cell therapy has shown promising preliminary results. Further clinical studies are warranted to confirm these observations.

### Role of GM-CSF in CRS

Granulocyte macrophage colony-stimulating factor (GM-CSF) is a crucial hematopoietic growth factor and regulator, implicated in processes such as hematopoiesis, inflammatory responses, infections, and enhancement of cytotoxic activity [54, 55]. This cytokine is secreted by a variety of cells, including monocytes, macrophages, T cells, B cells, fibroblasts, and epithelial cells [56]. Research has determined that CAR-T cells and T cells are the main sources of GM-CSF in patients with CRS after CAR-T therapy, and elevated serum GM-CSF levels are positively correlated with the severity of CRS [57, 58]. The mechanism of GM-CSF in CRS is complex, involving the interaction of multiple cell types and signaling pathways. Jiang Y and her colleagues observed that the pro-inflammatory effect of GM-CSF depends on the synergistic effect of other cytokines in the micro-environment, such as IL-6 and IL-8. When CAR-T cells are actively expanded, GM-CSF activates macrophages by binding to its receptor (CD116), inducing M1 polarization and promoting the release of pro-inflammatory cytokines such as IL-6, IL-1β, TNF-α, forming a positive feedback loop that exacerbates systemic inflammatory responses [59]. It is believed that this may be related to its involvement in the ERK1/2 and NF-κB pathways [60, 61]. But if CAR-T cell expansion decreases and lacks other synergistic factors, the use of GM-CSF alone may not trigger CRS [62]. GM-CSF is used to promote the reconstruction of myeloid cells after bone marrow transplantation, by enhancing the bone marrow production of neutrophils and monocytes, increasing the number of myeloid cells in peripheral blood [63].However, excessive GM-CSF may still exacerbate tissue inflammation. Overall, GM-CSF exhibits a double-edged sword effect in CAR-T therapy. Therefore, the optimal application window of GM-CSF in CAR-T therapy needs to be evaluated comprehensively based on cell dynamics and inflammatory markers, and further exploration is needed. In the treatment of CRS, whether through specific antibody therapy or TALEN mediated knockout of GM-CSF in CAR-T cells, the neutralization of GM-CSF is sufficient to reduce the release of inflammatory cytokines in vitro [57]. Lenzilumab is a GM-CSF neutralizing antibody that has been shown to reduce the toxicity of CAR-T cell therapy and improve treatment efficacy [58]. These findings highlight the potential of GM-CSF intervention as a strategy for managing CAR-T cell-related toxicity.

### Role of pyroptosis in CRS

**Fig. 2:**
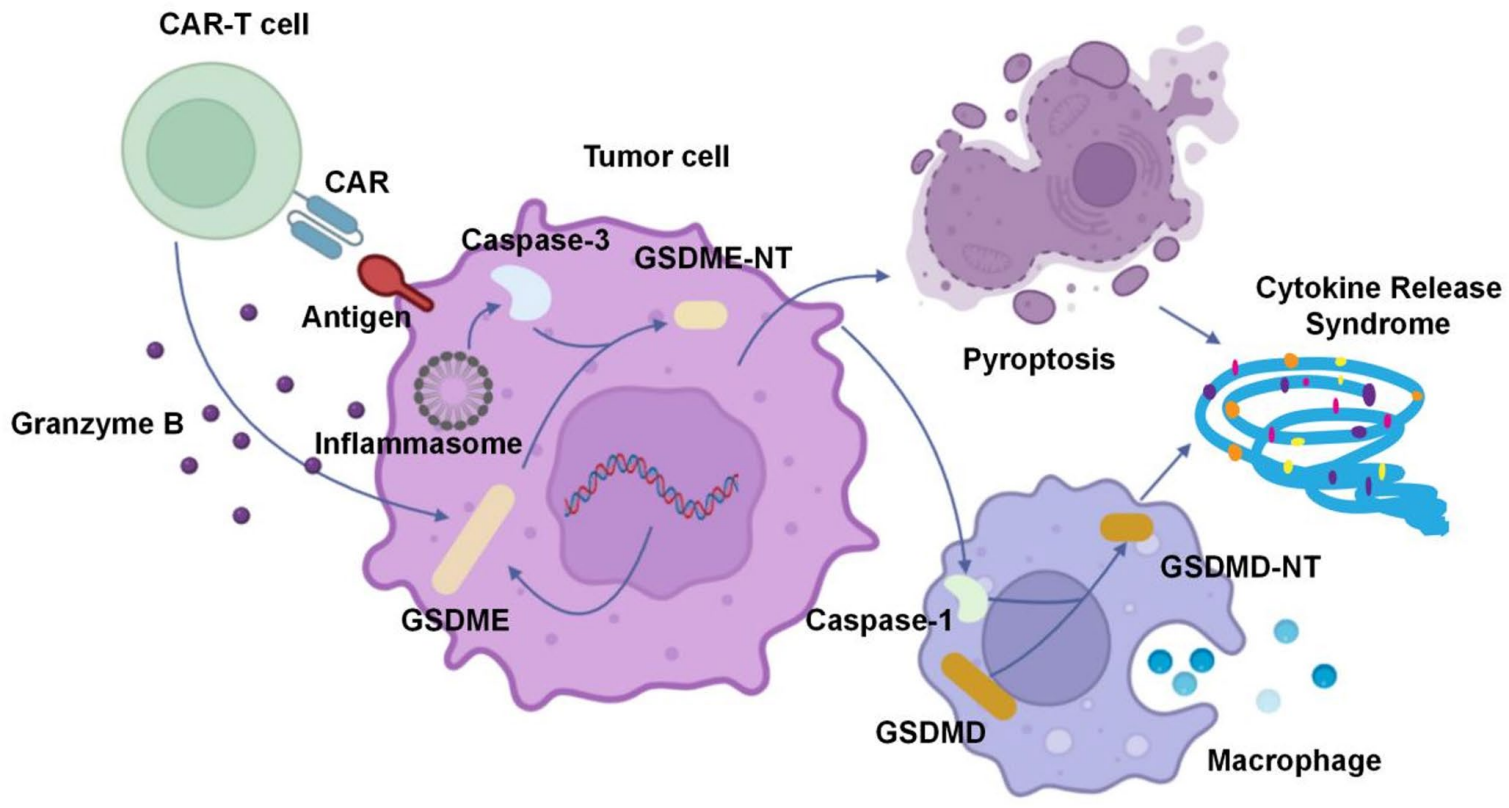
CAR-T cell-induced pyroptosis in target cells and subsequent macrophage activation. CAR-T cells release granzyme B, which activates caspase-3 in target cells (e.g., leukemia cells). Caspase-3 cleaves gasdermin E (GSDME) into GSDME-NT and triggers pyroptosis. Pyroptotic cells release DAMPs and cytokines which activate macrophages. Inflammasome-mediated caspase-1 activation in macrophages cleaves gasdermin D (GSDMD) into GSDMD-NT, amplifying pyroptosis and cytokine release (e.g., IL-1β, IL-6). This cascade contributes to cytokine release syndrome (CRS) and CAR-T-related toxicity. Key molecules involved are labeled in the diagram

Pyroptosis is a critical form of programmed cell death that plays a significant role in the immune response. It can be initiated by the activation of the caspase-1 inflammasome or by the cytoplasmic lipopolysaccharide-induced activation of caspases-4, 5, and 11 [64–66]. Recent research has indicated that CAR-T cells can trigger pyroptosis in target cells through the mediation of gasdermin E (GSDME). CAR-T cells rapidly activate caspase-3 in target cells by releasing granzyme B, and the latter cleaves GSDME, which is highly expressed in B leukemia cells and other target cells, leading to extensive pyroptosis [67–69]. This process subsequently activates caspase-1 in macrophages, leading to the cleavage of gasdermin D (GSDMD) and the release of cytokines, which can result in CRS. Studies have correlated higher levels of GSDME and lactate dehydrogenase with the severity of CRS in patients [68]. Macrophages are the main effector cells of pyroptosis. The IL-1β released after apoptosis activates the NF-κB pathway through IL-1R signaling, promoting the synthesis of cytokines such as TNF-α and IL-6, forming a positive feedback loop. During CAR-T therapy, excessive apoptosis of macrophages or activated T cells may lead to DAMP leakage. DAMPs are mainly produced or released by damaged and dying cells, and can trigger macrophage activation, leading to the development of inflammatory diseases [70–72]. Thus, further activation of the immune system and release of cytokines, elevated cytokine levels can induce tissue cell necrosis or pyroptosis, thereby forming a vicious cycle that exacerbates CAR-T related toxicity. Deng and colleagues emphasized the importance of DAMP released by monocytes/macrophages and pyroptosis cells as the main contributors and therapeutic targets of CAR-T related toxicity [73]. Therefore, strategies such as blocking apoptosis execution, consuming DAMP released by apoptotic cells, or improving CAR-T design to target tumor cell apoptosis without inducing apoptosis in healthy tissues are promising research directions for reducing CAR-T related toxicity. The specific form of Pyroptosis is shown in Fig. 2.

### Role of catecholamines in CRS

Catecholamines (CAs) are important stress hormones and neurotransmitters that regulate cardiovascular, metabolic, and immune systems by activating adrenergic receptors (α and β receptors) [74–76]. Recent research has highlighted the significant role of CAs in the development of CRS. In terms of immune regulation, it can activate α1-AR, activate macrophages and neutrophils, enhance the secretion of pro-inflammatory factors (such as IL-6, TNF-α), and the catecholamines secreted by macrophages can further act on their own or neighboring immune cell adrenergic receptors, forming a “self-amplifying circuit” that continuously promotes inflammatory response [77, 78]. High levels of IL-6 during CRS can directly activate the hypothalamic pituitary adrenal (HPA) axis and sympathetic nervous system, leading to sustained release of catecholamines and forming a vicious cycle of inflammation stress [76]. In B-ALL mouse models, inhibition of catecholamine synthesis by methyltyrosine (MTR) or atrial natriuretic peptide (ANP) resulted in a significant decrease in IL-6 and TNF-α levels and an improvement in survival rate without compromising the efficacy of CD19 CAR-T cells [78]. On the other hand, catecholamines enhance vascular constriction through α1-AR, while long-term high concentrations of catecholamines cause desensitization of β receptors, exacerbating vasodilation and hypotension. Meanwhile, IL-6 and TNF-α induce endothelial cell pyroptosis or apoptosis, disrupt the vascular barrier, and trigger fluid extravasation (such as pulmonary edema and acute respiratory distress syndrome, manifested as decreased blood volume and inadequate organ perfusion [77]. Finally, catecholamines can also cause metabolic disorders such as hyperactivity of glucose and lipid metabolism and lactic acidosis [76]. Metoprolol, a β1 adrenergic receptor blocker, has been shown to reduce IL-6 protein synthesis by inhibiting the eEF2K-eEF2-regulated translation extension [78]. A phase I/II clinical trial in non-Hodgkin’s lymphoma patients indicated that metoprolol is safe and effective in managing CRS, significantly alleviating CAR-T induced CRS without affecting the therapeutic efficacy of CAR-T cells [78]. These findings emphasize the potential of targeting the CA pathway in mitigating CRS associated with CAR-T cell therapies.

## Pathogenesis mechanisms of ICANS related to CAR-T cell therapies

**Fig. 3:**
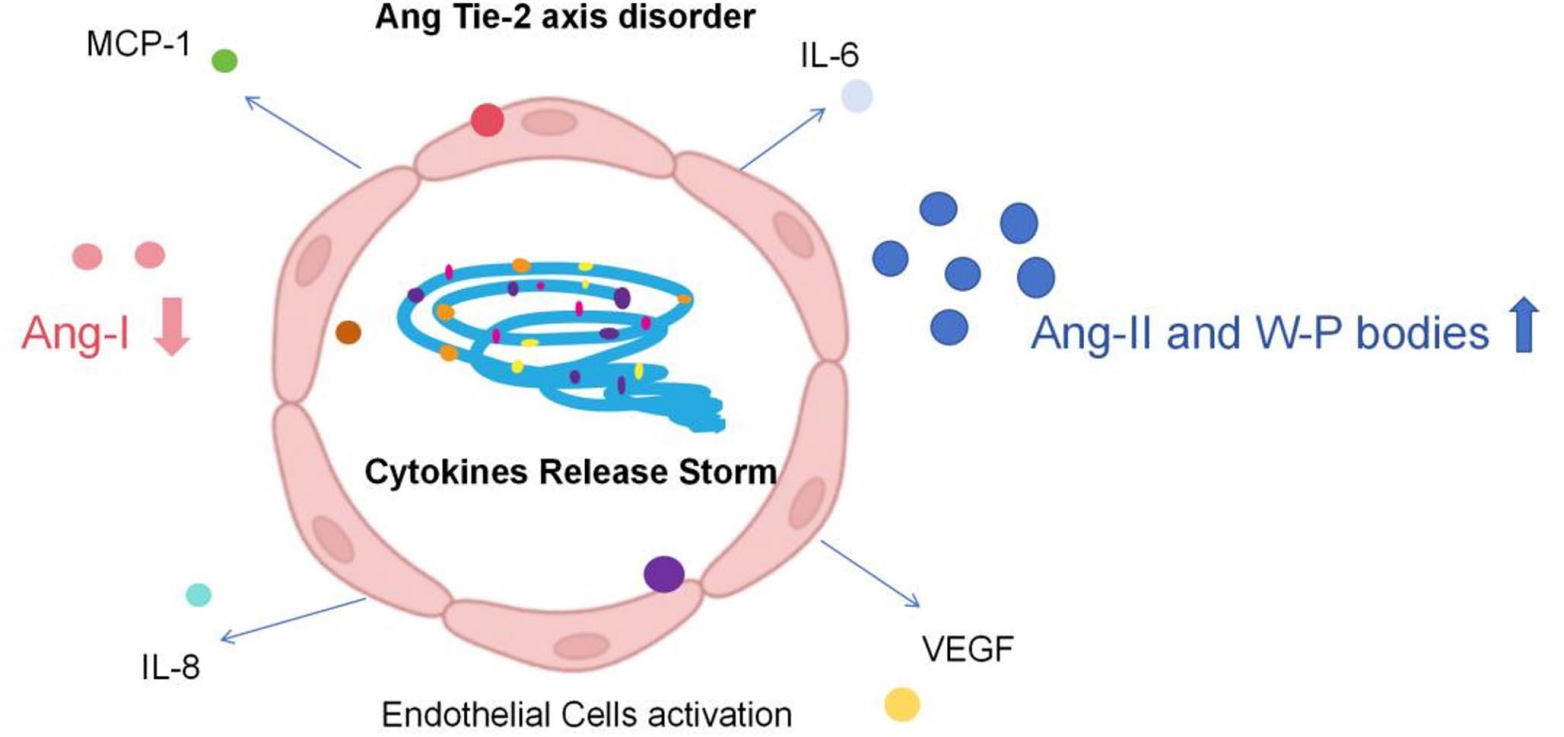
The mechanism of endothelial activation through Ang-2/Ang-1 imbalance and the release of cytokines (IL-6, VEGF) leads to blood-brain barrier disruption. Potential therapeutic interventions targeting MCP-1 or VEGF were identified

**Fig. 4:**
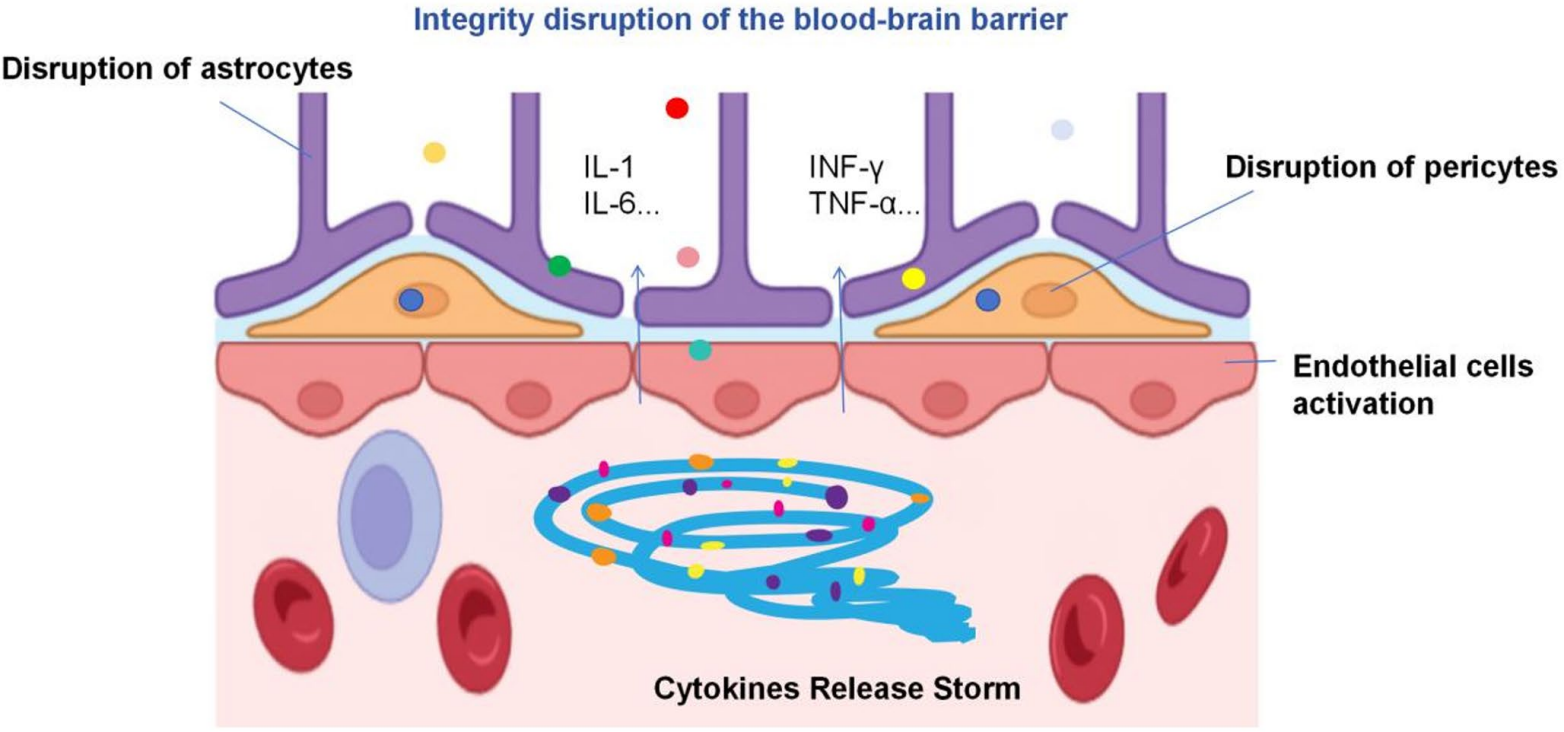
This figure illustrates the mechanism of blood-brain barrier integrity disruption in CAR-T associated neurotoxicity (ICANS). The dysfunction of astrocytes (affected by IL-1, IL-6, IFN - γ, TNF - α) and pericytes (affected by endothelial cell activation) leads to the invasion of inflammatory cells and cytokines into the central nervous system, causing neuroinflammation and neuronal damage

ICANS, refers to a series of clinical manifestations of neurological abnormalities caused by the activation or involvement of endogenous or exogenous T cells and/or other immune cells in patients after CAR-T therapy [79]. Severe CRS is a risk factor for the development of ICANS, which can occur simultaneously with CRS or in the following days. It often occurs 2–9 days after CAR-T infusion. Clinical manifestations of ICANS mainly include delirium, aphasia, tremor, writing disorders, drowsiness, epilepsy, cerebral edema, and coma [8]. ICANS can be graded by the 10-level scoring standard of Immune Effector Cell Associated Encephalopathy (ICE). On the basis of the ICE, the ICANS grading system also integrates the evaluation of clinical symptoms and signs across other neurological systems, including level of consciousness, severe motor weakness, seizures and elevated intracranial pressure or cerebral edema. Based on the five ranges of neurological assessment mentioned above, patients are classified according to any of the most severe symptoms [80]. The ICE score can be used as a daily screening and assessment of the risk period for ICANS attacks. The mechanism of ICANS is similar to the CRS, both involving toxicity caused by the secretion of various cytokines. The activation of endothelial cells and the disruption of the blood-brain barrier (BBB) are the core links of ICANS. The key pathogenesis mechanism of ICANS is shown in Figs. 3 and 4.

### Endothelial cell activation

Angiopoietin 1 and 2 (Ang-1 and Ang-2) are ligands of the Tie-2 receptor and have opposite functions in regulating endothelial activation. It is found that Ang-2 and von Willebrand factor are biomarkers of endothelial activation. Ang-1 tends to stabilize endothelial cells and inhibit pro-inflammatory pathways, while Ang-2 is released during the inflammatory process which can lead to endothelial cell activation and micro-vascular leakage [81]. The production of large number of cytokines after CAR-T infusion also promotes further inflammation and leads to endothelial cell activation. Activated endothelial cells secrete additional IL-6 and other pro-inflammatory factors, such as vascular endothelial growth factor (VEGF), IL-8, monocyte chemotactic protein-1 (MCP-1), which stimulates endothelial cells again and causes the release of W-P bodies and Ang-2 from endothelial cells [82]. Therefore, the increased Ang-2 in serum replaces Ang-1. The increase in Ang-2 and decrease in Ang-1 can inhibit the Tie-2 pathway and increase endothelial permeability, leading to dysfunction of the Ang-Tie-2 axis in balancing endothelial cell quiescence and activation [36]. It has been reported that the severity of ICANS is related to an increase in VWF, a decrease in Ang-1, an increase in Ang-2, and an increase in Ang-2: Ang-1 ratio [38]. As platelets are the main producers of Ang-1, thrombocytopenia may also be involved in the activation of endothelial cells. Therefore, micro-thrombus and disseminated intravascular coagulation may also lead to an increased incidence of ICANS.

### The disruption of Brain blood barrier

The brain blood barrier (BBB) plays an important role in maintaining the homeostasis of the central nervous system (CNS). However, massive cytokine release after CAR-T therapy can lead to endothelial activation, reversible contraction, impaired tight junctions, and even cell death, disrupting the integrity of BBB and activating coagulation [83]. Subsequently, CAR-T cells and other immune cells, as well as a large number of cytokines, migrate to the CNS and result in a significant increase in cytokine levels in the cerebrospinal fluid, such as IL-1, IFN-γ, TNF-α, and IL-6 [84]. The infiltrating immune cells along with activated pro-inflammatory cells exacerbate the inflammatory cascade reaction in CNS and lead to neuronal damage and dysfunction [8]. Other components of BBB, especially astrocytes and pericytes exacerbate inflammation and further damage the integrity of BBB when they expose to harmful cytokines [85]. CD19 is also expressed on the pericytes of the brain, indicating that targeting CD19 by CAR-T cells may also be a contributing factor in the pathophysiology of ICANS [28].

### Other factors in ICANS

Similar to CRS, the occurrence and development of ICANS are closely related to multiple cytokines, such as IL-1 and IL-6. For example, it has been demonstrated that monocyte-derived IL-1 and IL-6 are required for neurotoxicity by CAR-T cells and that targeted intervention against IL-1 and IL-6 may overcome neurotoxicity [86]. However, other cytokines such as IFN-γ, IL-10, GM-CSF, and IL-15 were uniquely elevated in patients with neurotoxicity, but not in patients with CRS alone. It has been reported that increased serum IFN-γ and IL-10 levels are associated with ICANS [87]. Significantly, although IL-10 may be as a counter-regulator that is important for maintaining an inflammatory response after CAR-T cell infusion, it could also lead to induction of additional toxic responses [87]. GM-CSF is also closely related to ICANS [86]. Compared to the incidence of CRS caused by GM-CSF intact CAR-T cells, the knockout of GM-CSF in CAR-T cells significantly reduced the incidence of ICANS [63]. This indicates that inhibiting GM-CSF may achieve the goal of preventing and treating ICANS. Several studies have shown that higher baseline level or peak level of serum IL-15 have been associated with better anti-tumor responses after anti-CD19 CAR-T therapy and higher rates of severe ICANS [88, 89].

## Pathogenesis mechanisms of HLH related to CAR-T cell therapies

Hemophilic lymphohistiocytosis (HLH) or macrophage activation syndrome (MAS) are clinical syndromes characterized by fever, hepatosplenomegaly, pancytopenia, and ferritemia, which was caused by pathological excessive inflammation and uncontrolled macrophage activation [90]. These immune regulatory abnormalities are mainly manifested by excessive activation of T lymphocytes, macrophages, as well as defects in normal cytolytic function of NK cells and CTLs, which together lead to a self-sustained state of excessive inflammation [91, 92]. Due to similar clinical manifestations, CRS and HLH are suspected to belong to a common spectrum. CAR-T related HLH often occurred with a higher-level systemic inflammatory response and a high mortality rate when traditional CRS has resolved [92]. The common laboratory abnormalities of HLH are similar to severe CRS, including ferritinemia, elevated CRP, hypofibrinogenemia and abnormal elevation of multiple cytokines [11]. Therefore, the diagnosis of HLH can be difficult in the context of CRS. HLH is defined as SF > 10,000 µg/L, accompanied by ≥ 3 levels of organ toxicity (liver, kidney, lungs), and hemophobias may be seen in the bone marrow or other organs. It is worth noting that compared with CRS patients with IL-6 as the core, HLH patients treated with CAR-T showed significant and sustained increases in IFN-γ and IL-1 [10, 93].

### Role of IFN-γ in HLH

It is known that NK cell activity can limit CD8 + T cell immunity [94]. In HLH patients, NK cells and CTLs have defects in cytolytic function, which may lead to uncontrolled CAR-T cell proliferation and expansion, resulting in excessive secretion of IFN-γ, which is considered as a key cytokine driving HLH [16]. IFN-γ activates the JAK-STAT signaling pathway, induces macrophages to secrete pro-inflammatory factors such as IL-1β and IL-18. Abnormally activated macrophages are also a characteristic of HLH. They can also secrete IFN-γ, which can reactivate the monocyte macrophage system and secrete large amounts of cytokines [92]. This large amount of cytokines promotes hemophagy. Preclinical studies suggested that IFN-γ blockade emapalumab could reduce macrophage activation and cytokine production. In relevant clinical studies, IFN-γ increases disproportionately in HLH patients and emapalumab has shown efficacy in treating HLH [16, 91, 95]. However, more clinical data are needed to verify the efficacy of IFN-γ blockade in the management of HLH.

### Role of IL-1 in HLH

IL-1 levels were particularly high among those HLH, patients which may be related to the secretion of more IL-1 by abnormal activation macrophages, and HLH has a good clinical response to IL-1 blockade [10, 92, 96]. Anakinra is a recombinant IL-1 receptor antagonist, and it has been shown in some case reports to be a viable alternative to initial treatment for secondary HLH patients with or without dexamethasone. Intravenous anakinra has a wide range of therapeutic doses and tolerability, demonstrating significant efficacy in severe HLH regardless of the triggering factors. It is recommended to start using anakinra as early as possible when HLH is suspected [97, 98]. More extensive research is currently underway on the use of anakinra as a first-line drug for HLH.

## Management of CRS, ICANS, and HLH related to CAR-T cell therapies

**Fig. 5:**
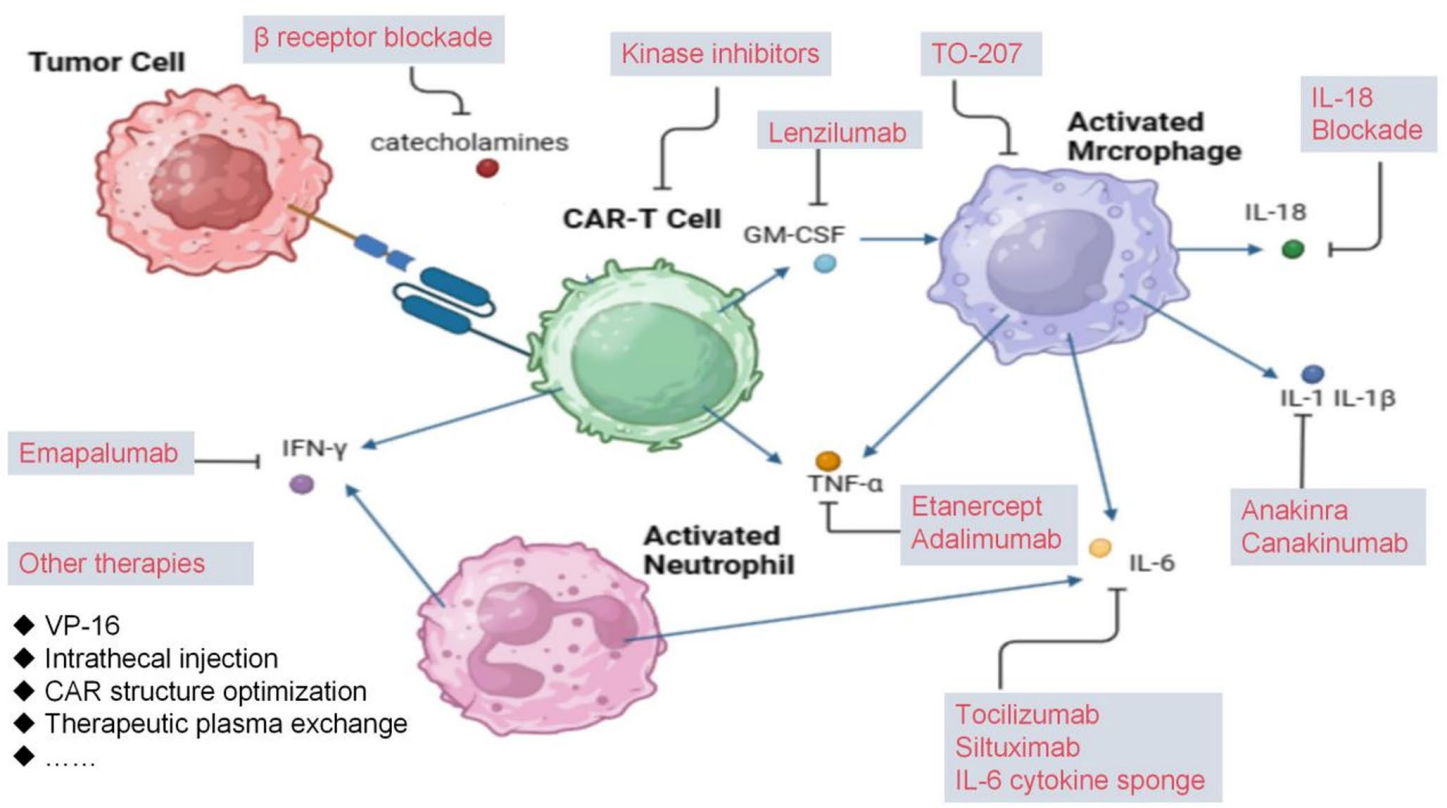
This figure provides a comprehensive overview of therapeutic interventions for CAR-T cell therapy-associated toxicities. The schematic highlights three principal therapeutic approaches targeting different pathological mechanisms. Cytokine-directed therapies form the cornerstone of management, with agents such as tocilizumab (IL-6 receptor antagonist), anakinra (IL-1 receptor antagonist), lenzilumab (GM-CSF neutralizing antibody), and specific cytokine blockers including etanercept (TNF-α inhibitor) and emapalumab (IFN-γ monoclonal antibody). Small molecule inhibitors like ruxolitinib (JAK1/2 inhibitor) and dasatinib (SRC kinase inhibitor) modulate downstream inflammatory signaling pathways. Supportive measures including therapeutic plasma exchange, etoposide (VP-16) for HLH, and intrathecal corticosteroids for neurotoxicity management complete the multidimensional treatment strategy

CRS, ICANS, and HLH are caused by excessive cytokines release following CAR-T cell infusion. As a result, they share similarities in the management of these conditions. However, there are some differences in their pathogenesis that lead to somewhat different treatments in these cases. Crucial treatment strategies are shown in Fig. 5.

### Corticosteroids

Corticosteroids are an effective treatment method for inhibiting excessive inflammatory response, they produce many anti-inflammatory effectors on T cells, monocyte/macrophages and cytokines [99, 100]. At present, corticosteroids are widely used in clinical practice for the treatment of CRS, ICANS, and HLH. Different dosage is selected according to the severity of the condition [15, 38, 101]. Research has shown that preventive use of corticosteroids can reduce the incidence and mortality of CRS, ICANS, and HLH [15, 18, 101]. Corticosteroids can induce systemic immune suppression, inhibit the proliferation of immune cells and the production of cytokines, effectively inhibit toxicity and alleviate symptoms in patients. It has been found that corticosteroids can prevent the progression of CRS in B-cell line acute lymphoblastic leukemia patients early and timely through early preventive intervention [102]. High dose steroids are the cornerstone of treating HLH, and the administration of corticosteroids alone can effectively prevent or inhibit inflammation in mild HLH [103]. Corticosteroids are an effective non-specific anti-inflammatory treatment option, but some reports have also shown the ablation effect of steroids on CAR-T cells. A prospective clinical trial found that tocilizumab treatment for CRS did not affect CAR-T cell expansion, while corticosteroids caused CAR-T cell regression [14, 104]. Some evidence suggests that corticosteroids may inhibit the persistence of CAR-T cells and the efficacy against malignant tumors, as previously reported in ALL patients after CD19 CAR-T cell infusion [23]. A retrospective analysis showed that higher cumulative doses of corticosteroids were associated with significantly shorter progression survival (PFS) and overall survival (OS) [105]. However, clinical trials have shown that using short-term steroid therapy for recurrent/refractory B-ALL patients after CAR-T treatment can achieve good control of CRS while not affecting CAR-T efficacy [106]. Therefore, it is currently recommended to use the lowest effective dose and shortest effective duration of steroid drugs to treat side effects after CAR-T. Due to the effectiveness of tocilizumab, corticosteroids are only considered as a second-line treatment for CAR-T cell-induced CRS. Methylprednisolone or dexamethasone are commonly used in clinical practice. Dexamethasone has a higher penetration efficiency through the blood-brain barrier, so it can also be used for intrathecal therapy mentioned below to reduce neurotoxicity. For patients who use steroids for a long time in clinical practice, it is inevitable that there is a risk of multiple infections. Therefore, preventive measures are often used in combination with various antibacterial, antifungal, and antiviral drugs to reduce the occurrence of infections and worsen the condition in patients [107].

### IL-6/IL-6 receptor (IL-6R) blockade

IL-6, as a key cytokine in the occurrence of CRS, can be effectively blocked by tocilizumab by blocking the IL-6 signal [24]. The IL-6 receptor antagonist tocilizumab is a first-line cytokine therapy approved by the US Food and Drug Administration for CRS [14]. It can quickly reverse the symptoms of CRS and has no significant adverse effects on the immune system. It has been found that 46% of patients with relapsed/refractory acute lymphoblastic leukemia had grade 3 to 4 CRS after CAR-T cell therapy, but the incidence rate of CRS was significantly reduced after using tocilizumab [108]. In addition, no adverse events related to the administration of tocilizumab were reported in the evaluation of CAR-T related CRS patients treated with tocilizumab in retrospective analyses of multiple clinical studies [14]. Research has shown that tocilizumab is ineffective in treating CAR-T neurotoxicity in the mouse model [28]. This is because tocilizumab blocks IL-6R and reduces the clearance rate of IL-6, leading to a brief increase in serum IL-6. Additionally, tocilizumab is unable to cross the blood-brain barrier [109], further increasing the concentration of IL-6 in cerebrospinal fluid. Therefore, it is believed that the use of tocilizumab may even exacerbate ICANS [110]. However, in the early stage of severe CRS, the concentration of serum inflammatory cytokines significantly increases, which is the most important risk factor for the occurrence of ICANS [109]. Therefore, some scholars speculate that preventing severe CRS through preventive measures can reduce the incidence of ICANS. The study found that the prophylactic use of tocilizumab 1 h before CAR-T infusion in 20 patients with relapsed/refractory lymphoma who were about to receive CD19 CAR-T cell therapy was related to the low incidence rate and severity of CRS/ICANS, and there was no adverse event related to tocilizumab [111]. At present, the combination of tocilizumab and corticosteroids is widely used in the treatment of CRS after CAR-T, but when ICANS and CRS occur simultaneously, CRS is usually prioritized and tocilizumab should be used with caution.

Well, siltuximab may be a substitute drug for tocilizumab to clear IL-6 from the cycle by combining IL-6, thereby reducing it into the CNS and alleviating ICANS [26]. Siltuximab has a higher affinity for IL-6 than tocilizumab, so patients who do not respond to tocilizumab and corticosteroid may consider using situximab. Chen et al. showed that situximab alone is effective and safe in treating CRS through a comparative study of four patients [112]. On the other hand, Abboud et al. found that the use of tocilizumab and situximab alone or in combination can reduce CRS in a cohort study of 75 patients, even in the most severe cases [113]. Therefore, siltuximab can also be used in combination with tocilizumab to prevent or treat severe CRS and neurotoxicity [114].

Recently, A research group proposed the “cytokine sponge” strategy to prevent CAR-T cell-induced CRS [115]. The researchers chemically linked the IL-6 antibody to the thermosensitive hydrogel, and injected the IL-6 antibody hydrogel conjugate (cytokine sponge) under the skin before the CAR-T cells were transfused back. When CAR-T cells induce an inflammatory cytokine storm, the injected cytokine sponge can adsorb the inflammatory cytokine IL-6 in real-time and controllably based on the concentration of IL-6 to prevent and inhibit CRS related symptoms. This responsive adsorption characteristic shifts the strategy of CRS management from traditional antibody blockade and relief to effective prevention and inhibition. The team has validated on various animal models that “cytokine sponge” not only effectively inhibits the elevation of various inflammatory factors, alleviates CRS related symptoms, improves survival rate, but also does not affect the anti-tumor effect of CAR-T. Currently, this mechanism is still under research and has not been applied in clinical treatment.

### IL-1 receptor (IL-1R) blockade

Due to the significant increase of IL-1 in CRS, ICANS, and HLH, IL-1 can also be a key target for treatment. It has been found that the IL-1 receptor antagonist anakinra reduced CRS related mortality through in vivo experiments in mice [28]. Both IL-1 and IL-6 blockade resulted in a similar decrease in iNOS + macrophage components [116]. Therefore, downregulation of iNOS was established as a unified mechanism by which IL-6 and IL-1 blockade can alleviate CRS. The Sadelain’s team has also demonstrated that prophylactic use of the IL-1 receptor antagonist anakinra can significantly reduce the incidence of ICANS caused by CD19 CAR-T cell therapy, without affecting the therapeutic effect of CAR-T cells [116]. A retrospective study analyzed clinical and laboratory parameters of 14 patients who developed steroid resistant ICANS with or without CRS after CAR-T therapy, and researchers observed significant and rapid reductions in fever, inflammatory cytokines, and ICANS/CRS related biomarkers after treatment with anakinra. This suggests that the IL-1R antagonist anakinra may be a useful adjunct to the treatment of steroid refractory CRS and/or ICANS caused by CAR-T cell therapy with steroids and tocilizumab [117]. Multiple retrospective studies have analyzed the prognosis of malignant tumor patients with refractory CRS or ICANS after CAR-T therapy, and the safety and efficacy of anakinra have been validated in these patients [114, 118]. It can be used in combination with tocilizumab to prevent severe cytokine release and neurotoxicity [112]. HLH and CRS also have significant overlap in pathophysiology, and some studies and case reports have shown that anakinra exhibits good therapeutic effects on HLH [119]. According to reports, HLH showed a significant improvement in response to anakinra after insufficient response to glucocorticoids [120, 121]. In vitro experiments have shown that the monoclonal antibody Canakinumab targeting IL-1β also has therapeutic potential for CAR-T related CRS and neurotoxicity [29, 40], but there is currently no relevant clinical trial data available.

### Kinase inhibitor

Elevated cytokines in CRS, ICANS, and HLH, such as IFN-γ, IL-2, IL-6, IL-10, and GM-CSF, signal through pathways involving Janus kinase (JAK) and transcription activating factor (STAT) [122]. Interrupting the JAK-STAT pathway by inhibiting signal transduction downstream of many related cytokines is expected to more effectively alleviate the pathophysiology associated with CRS, ICANS, and HLH. Currently, many JAK inhibitors, such as ruxolitinib, tofacitinib, and octacitinib, which have been used to treat inflammatory diseases [123]. Common JAK inhibitors, including ruxolitinib that blocks JAK1 and JAK2, or itacitinib that blocks JAK1, are expected to block the action of pro-inflammatory cytokines such as IFN-γ and IL-6. In fact, in preclinical models of CAR-T cell related toxicity, ruxolitinib and itacitinib can reduce toxicity and cytokine secretion, and does not affect the anti-tumor activity of CAR-T cells [124, 125]. Researchers used ruxolitinib to treat wild-type mouse models of HLH, demonstrating that ruxolitinib reversed many HLH manifestations, including splenomegaly, decreased blood cells, hypercytosis, peripheral organ and CNS inflammation, and significantly prolonged survival [126, 127]. Summarizing the literature on the use of ruxolitinib in HLH patients, it can be found that patients’ clinical manifestations can be released within 24–48 h after medication, such as fever, shock, renal failure, and respiratory depression. In addition, serum ferritin, soluble IL-2 receptor, and fibrinogen levels usually return to normal within 7–30 days [128]. Therefore, ruxolitinib or other JAK inhibitors can be proven to help improve the signs and symptoms of immunotherapy related CRS, ICANS, and HLH. In addition, the combination of ruxolitinib and glucocorticoids, the combination of ruxolitinib and HLH-1994 regimen, or the combination with DEP regimen can also be used as salvage treatment for refractory HLH patients. The combination use of ruxolitinib and other medication, including tocilizumab or low-dose glucocorticoids, may be beneficial for improving the management of side effects after CAR-T treatment [125]. Our center also found that after the combined treatment of CRS, ICANS, and HLH patients with ruxolitinib, cytokines were significantly reduced, resulting in significant benefits for the patient.

TKI can target and control specific signaling pathways within CAR-T cells, reducing the severity of CRS and ICANS. Mestermann et al. demonstrated that dasatinib can transform CAR-T cells into a functionally closed state in vitro and mouse models, reversibly inhibiting CAR-T cell proliferation and cytotoxicity. Dasatinib can quickly and reversibly inhibit CAR T cell-mediated cytotoxicity and cytokine production by targeting SRC kinase. In preclinical models, short-term administration of dasatinib can reduce CRS related mortality and does not impair in vivo anti-tumor effects [129]. Other SRC inhibitors of TKI, such as ponatinib and sarabatinib, can also inhibit the cytotoxic activity of CAR-T cells. FLT3 inhibits TKI, midostaurin can inhibit CAR cell toxicity by interfering with FLT3 mediated signaling [130]. In addition, ibrutinib, a Bruton’s tyrosine kinase (BTK) inhibitor, can inhibit the IL-2 induced tyrosine kinase (ITK) pathway and decrease the release of various cytokines [131–133]. Research has shown that the simultaneous use of ibrutinib and CAR T cells in the treatment of CLL can exert a synergistic anti-tumor effect while improving CRS [134]. Similarly, the BTK inhibitor erlutinib leads to a decrease in cytokine secretion, but studies have shown that it will weaken CAR-T cell activation [134]. Therefore, the clinical use of BTK inhibitors still requires more clinical validation.

### Therapeutic plasma exchange

Therapeutic plasma exchange (TPE) refers to an in vitro technique performed in a separation device to separate and remove a patient’s plasma from whole blood, while infusing blood cells with a substitute liquid back into the patient’s body [135]. Studies have shown that plasma exchange, as a broad-spectrum and non-specific method, has shown significant effectiveness in treating cytokine storms in recent years [136, 137]. Plasma exchange has ability to regulate systemic inflammation and coagulation function. It can remove cytokines from the blood in a non-targeted manner, regulate overactivated immune responses in patients, and promote immune reconstitution [138]. For grade 3–4 CRS or severe ICANS and HLH that are ineffective in tocilizumab treatment, it is recommended to use auxiliary measures such as hemodialysis filtration and plasma exchange in clinical practice to save time for patient treatment. A clinical study of 17 patients from three centers showed that patients with corticosteroid and tocilizumab resistant CRS with or without ICANS after CAR-T treatment showed significant improvement in clinical symptoms and laboratory indicators after TPE treatment. The rapid decrease in temperature and cytokine index levels indicated that TPE had a significant clearing effect and high safety on cytokine storms that were ineffective with corticosteroids and tocilizumab after CAR-T treatment [139]. Bosnak M reported two children diagnosed with HLH, both of whom received the HLH-2004 regimen with poor efficacy. After receiving multiple therapeutic plasma exchanges (TPE), the condition of both patients improved compared to before. By describing these two cases, it is emphasized that TPE can produce rapid improvement before stem cell transplantation in HLH patients who have not responded to traditional treatment [140]. At the same time, TPE can improve the coagulation function of most diseases, but coagulation dysfunction can also occur due to the loss of coagulation factors and platelets during plasma exchange. Therefore, monitoring the coagulation dynamics of patients during plasma exchange is crucial to ensure that the treatment is safer and more effective.

### Intrathecal corticosteroids or chemotherapy

If central nervous system symptoms such as epilepsy or seizures occur simultaneously with CRS, intrathecal injection of corticosteroids should be performed as soon as possible. Currently, corticosteroids are widely used for ICANS treatment in clinical practice, but the higher the cumulative dose of hormones, the worse the progression free survival (PFS) and overall survival (OS) of patients [105]. However, a study showed that early intrathecal injection of corticosteroids or combined with chemotherapy for ICANS treatment could help shorten the exposure time and cumulative dose of steroids, and prolong the patient’s PFS [141]. Katsin M reported a patient with stage IV primary mediastinal B-cell lymphoma who received methotrexate, cytarabine, and dexamethasone as first-line treatment for CD19 CAR-T cell related grade IV ICANS. They found that ICANS decreased to grade 0 steadily and rapidly after intrathecal injection of corticosteroids, so the use of systemic corticosteroids could also be stopped to avoid CAR-T cell ablation and ensure the preservation of CAR-T cell function [142]. According to reports, ICANS in five children and young patients with relapsed/refractory B-cell acute lymphoblastic leukemia who received systemic steroid treatment continued to worsen, but the symptoms rapidly reversed after intrathecal injection of hydrocortisone treatment [143]. Therapeutic lumbar puncture has shown significant safety and efficacy in some case reports, but the number of cases is relatively small and more clinical research data is still needed to supplement and support it.

In summary, whether it is lymphoma or leukemia, especially acute lymphocytic leukemia, central invasion is more likely to occur. Intrathecal therapy may be a valuable central nervous system targeted therapy for the treatment of high-grade or steroid refractory ICANS, it can reduce systemic immune suppression and treatment-related mortality associated with CAR-T cell therapy. Currently, chemotherapy drugs such as cytarabine, methotrexate, and dexamethasone are commonly used in clinical practice for injection treatment. If patients have central invasion, it plays a therapeutic role. If there is no central invasion, we suppose that it has a preventive effect, so this approach can also alleviate ICANS symptoms or prevent the occurrence of ICANS. Similarly, we recommend that for patients with HLH after CAR-T, if diagnosed with CNS-HLH, intrathecal injection of methotrexate and dexamethasone should be given as early as possible if the condition permits.

### Etoposide or VP-16

Etoposide (ETO) is a potent topoisomerase II toxin that can cause double stranded DNA breakage. If not repaired, it can lead to cell death. It is also a genotoxic compound that occasionally causes serious side effects and secondary leukemia [144]. In addition to its well-known function of inducing cancer cell death, ETO can also be used to treat immune mediated inflammatory diseases associated with cytokine storm syndrome [145]. ETO is essentially an immune modulator that can counteract excessive activation of CD8 + T cells and reduce the production of pro-inflammatory signals, such as IL-6, IL-10, and IL-18, which helps alleviate persistent inflammation in HLH patients [146, 147]. The combination of etoposide and glucocorticoids is the standard regimen for treating all types of HLH by targeting overactivated lymphocytes and macrophages. At present, for patients with HLH after CAR-T treatment, the most commonly used initial immunotherapy and chemoimmunotherapy regimens are the HLH-1994 regimen or HLH-2004 regimen (etoposide + dexamethasone, combined or not combined with cyclosporine induction therapy). Recently, data has shown that early use of etoposide based HLH treatment can reverse fatal outcomes in critical patients and achieve similar survival outcomes in non-critical HLH patients, and some patients even have the opportunity to achieve clinical cure [148]. Therefore, early identification and diagnosis of severe HLH, as well as early use of the DEP regimen for refractory HLH, are beneficial for improving the clinical outcomes of patients with HLH.

### Metoprolol and inhibitors of catecholamine synthesis

Metoprolol is a β1 adrenergic receptor blocker that exerts pharmacological effects by competitively binding to β1 adrenergic receptors and their endogenous ligand catecholamines. Metoprolol has the effects of slowing heart rate, lowering blood pressure, reducing myocardial oxygen consumption, controlling the development of angina, and improving the prognosis of heart failure. Clinical observations have shown that metoprolol is beneficial for improving CAR-T induced CRS. After clinical observation, Han W further investigated the mechanism using patient samples and human primary cells and found that metoprolol alleviated CRS by inhibiting the translation of IL-6 protein molecules in human monocytes [78]. Metoprolol works by inhibiting the protein translation extension of IL-6 in monocytes and macrophages, and first illuminated the possibility of protein translation as a therapeutic target for controlling CRS and the molecular mechanism by which metoprolol inhibits IL-6 translation. In addition, in a study by Staedtke, it was found that self-amplifying catecholamine rings in macrophages play a crucial role in the pathogenesis of CRS. Early use of tyrosine hydroxylase inhibitor metyrosine (MTR) or atrial natriuretic peptide (ANP) can prevent CRS caused by CD19 CAR-T cell infusion by reducing the production of catecholamines and various cytokines, without affecting the efficacy of CAR-T cells [77]. However, there are no clinical data reports on catecholamine synthesis inhibitors, and further clinical validation is still needed.

### TNF-α blockade

Recently, there is evidence to suggest that the occurrence of CRS and ICANS is closely related to the activation of vascular endothelial cells [35, 36, 38]. TNF-α is one of main mediators of endothelial activation in the cytokines produced by the participating CAR-T cells themselves and in the myeloid cells activated by CAR-T therapy, and are potential targets for CAR-T related CRS and neurotoxic cytokine therapy. The in vitro experiments conducted by Chen Y and colleagues also showed that the combination of TNF-α antibody adalimumab and anti-IL-1β antibody simultaneously can exert a synergistic effect in preventing endothelial cell activation, suggesting that blocking TNF-α and IL-1β may have therapeutic potential for CAR-T related CRS and ICANS [40]. It has been found that some patients with multiple myeloma had significantly elevated levels of TNF-α during CRS after BCMA CAR T cell infusion. By using the TNF-α inhibitor etanercept, three patients were successfully cured of CRS. Not only did they not change their response to CAR-T cell therapy, but no adverse events directly related to etanercept administration were observed [53]. However, due to the limited number of related clinical cases, further clinical validation is still needed.

### IFN-γ blockade

IFN-γ is considered a key cytokine driving HLH, and the increase in IFN-γ is also associated with severe CRS and ICANS. Therefore, in the treatment of refractory and life-threatening complications, IFN-γ may be a potential target [16]. Emapalumab is a monoclonal antibody targeting IFN-γ and was the first approved targeted therapy for the treatment of HLH [149], but currently there is no clinical application of emapalumab for the treatment of CRS/ICANS. On the one hand, a phase II/III clinical study analyzed the efficacy and safety of emapalumab administered together with dexamethasone. It has been found that emapalumab is an effective targeted therapeutic drug which can inhibit CAR-T cell related toxicity, thereby improving symptoms and signs of HLH [150, 151]. A prospective single arm trial included 14 refractory HLH/MAS patients who did not respond to high-dose corticosteroid treatment (with or without combination with anakinra), and all clinical and laboratory parameters showed rapid improvement after receiving anti IFN-γ treatment [152]. By week 8, 13 out of 14 patients achieved remission within a median of 25 days [152]. However, Fajgenbaum believed that emapalumab may pose significant risks to the efficacy of CAR-T cells [153]. Therefore, the impact of emapalumab on the efficacy of CAR-T is still controversial, which may be related to drug dosage and optimal administration time. More data are still needed to explore the clinical application of IFN-γ monoclonal antibodies in CRS or ICANS.

### GM-CSF Blockade

Lenzilumab is a neutralizing antibody against human GM-CSF. Lenzilumab can target and neutralize the activity of GM-CSF, inhibit the inflammatory cascade initiated by GM-CSF, and prevent and treat cytokine storms and neurotoxicity related to GM-CSF during CAR-T treatment [154]. After neutralizing GM-CSF with lenzilumab, Sterner found that CD19 CAR-T cell proliferation was enhanced and sustained control of leukemia was better maintained in patient derived xenograft models, demonstrating that lenzilumab can not only enhance CD19 CAR-T cell function in vitro or in vivo, but also prevent and treat CRS and ICANS [58]. It has been reported that lenzilumab could address the ICANS induced by CAR-T cells in a mouse model by inhibiting the entry of myeloid cells and T cells into the CNS [79]. However, there is currently no relevant experimental data applied to humans.

### Other prevention and management of toxicities

Calcineurin is a protein phosphatase that plays a role in T cell activation, and tacrolimus and cyclosporine can inhibit the activity of calcineurin. Studies have shown that calcineurin inhibitor can interfere with the signal transduction process of CAR T cells, thereby inhibiting their cytotoxic activity and alleviating CRS or ICANS [155]. At present, tacrolimus is mainly used to prevent graft-versus-host disease (GVHD) in clinical practice, its therapeutic effect in CRS or ICANS still needs further exploration. Mammalian target of rapamycin (mTOR) inhibitors can reduce T cell proliferation and cytokine release after TCR activation through CD3 stimulation [156]. Currently, clinical mTOR inhibitors such as sirolimus can prevent T cell-mediated cytokine release while still retaining the killing effect of CAR-T therapy. Therefore, targeting the mTOR pathway may alleviate inflammation driven adverse events related to immunotherapy while maintaining its efficacy.

Due to the pro-inflammatory factor IL-18 can induce cells to produce other inflammatory cytokines, the pro-inflammatory effect of IL-18 can be neutralized by high affinity endogenous IL-18 antagonist binding proteins. Therefore, IL-18 has become an attractive therapeutic target [157]. When using IL-18 receptor monoclonal antibodies, it can serve as a therapeutic option to prevent symptoms such as pancytopenia and hemophilia syndrome associated with HLH. There is currently no approved treatment method specifically designed to reduce IL-18 activity. However, by utilizing the high affinity of IL-18 binding protein (IL-18BP) for IL-18, recombinant human IL-18BP (rhIL-18BP) is undergoing clinical trials for secondary HLH [157], and further research and detailed validation are still needed.

It has been found that the multi cytokine inhibitor TO-207 could inhibit the production of various cytokines derived from monocytes such as TNF-α, IL-6, IL-18, GM-CSF. It can inhibit mRNA maturation without affecting the cytotoxicity of CAR T cells [158], thereby alleviating symptoms of CRS, ICANS, and HLH. Wei Y found that pre-treatment with THZ1 to block cyclin dependent kinase 7 (CDK7) can inhibit inflammatory genes, especially STAT1 and IL-1, at the transcriptional level. Therefore, it can alleviate cytokine toxicity without compromising anti-tumor efficacy [159].

Optimizing the design of CAR molecules can also significantly affect the proliferation of CAR-T cells and the distribution of cytokines, thereby reducing the incidence and severity of CRS or ICANS [160].

## Discussion

**Fig. 6:**
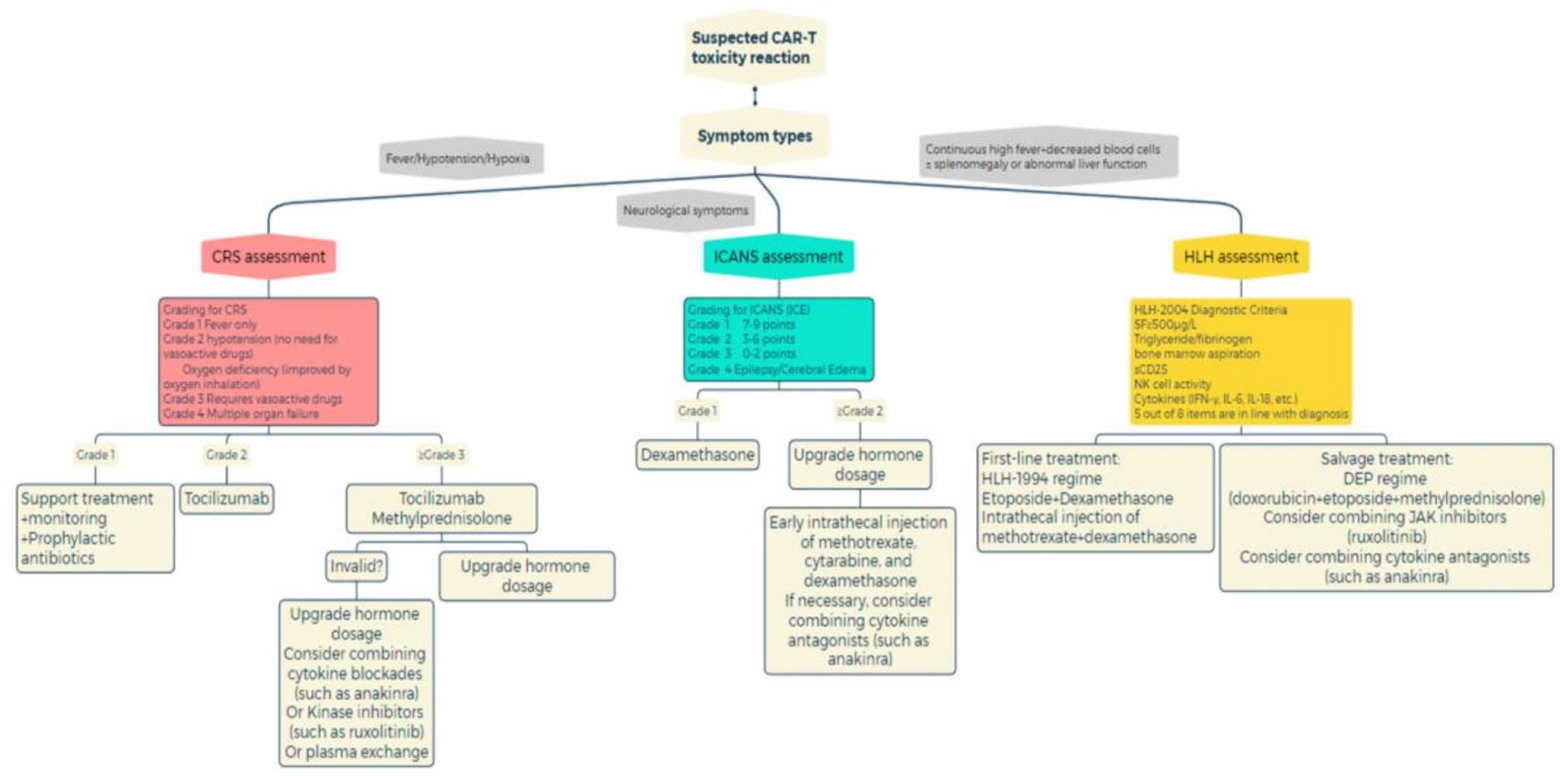
This flowchart outlines the assessment and treatment strategies for three major CAR-T therapy-related toxicities: CRS, ICANS, and HLH. CRS grading and intervention: Grade 1: supportive care (such as prophylactic antibiotics). Grade 2: tocilizumab. Grade 3/4: tocilizumab + methylprednisolone. For refractory cases, steroid dosage gradually increases. ICANS grading and intervention: Grade 1/2: dexamethasone. Grade 3/4: high dose steroids, intrathecal injection of methotrexate and dexamethasone

The advent of CAR-T cell therapy has revolutionized the treatment of hematologic malignancies, yet its clinical application is significantly limited by the associated toxicities, including cytokine release syndrome (CRS), immune effector cell-associated neurotoxicity syndrome (ICANS), and hemophagocytic lymphohistiocytosis (HLH). Safarzadeh’s retrospective article focuses on the clinical strategies for managing CRS and ICANS, including the application of some blockades, antibodies, and plasma exchange, as well as the clinical efficacy provided in relatively limited studies at the time, with relatively little description of toxicity mechanisms [161]. On this basis, this review has added toxicity reactions to HLH, providing a detailed summary of the latest insights into the pathogenesis and management strategies of these complications, highlighting both established and emerging therapeutic strategies. A simple flowchart summarizing the strategies for managing CAR-T toxicity is shown in Fig. 6. We systematically summarized the clinical efficacy and safety of different treatment strategies for CRS, ICANS, and HLH induced by CAR-T therapy in preclinical or clinical trials in Table 1.

HLH diagnostic criteria: HLH-2004. First line treatment: HLH-1994 regime (etoposide + dexamethasone, Intrathecal injection of methotrexate + dexamethasone). Remedial measures: DEP regimen (doxorubicin + etoposide + methylprednisolone) ± JAK inhibitors (such as ruxolitinib) or cytokine blockades (such as anakinra).

**Table 1:** Clinical efficacy and safety of different treatment strategies in preclinical or clinical trials

Strategy type	Sample size	Clinical trial phase/Case report	Patient response rate	Major adverse effects	References
IL-6 Inhibition					
Tocilizumab (IL-6R mAb)	45 patients (24 men and 21 women)	FDA-approved	53–69% responded (CRS G3-4)	No adverse events	Le et al. 2018 [14]
Prophylactic use of tocilizumab	20 patients: 18 locally manufactured CD19 CAR-T 2 tisagenlecleucel	/	No cases of CRS ≥ G3, ICANS G4 5%	No adverse events	Caimi et al. 2021 [111]
IL-1 Inhibition					
Anakinra (IL-1Ra)	43 patients: 21 administered subcutaneously 20 intravenously 2 both	/	Higher anakinra dose may be associated with faster CRS/ICANS resolution	Infection and liver dysfunction risk (6%)	Gazeau et al. 2023 [118]
Prophylactic use of anakinra	31patients: 74% axicabtagene ciloleucel, 13% brexucabtagene ciloleucel 4% tisagenlecleucel	Phase II (NCT04148430)	All grade ICANS 19% ≥ ICANS G3 9.7%	No adverse events	Park et al. 2023 [116]
Canakinumab (anti-IL-1β)	/	Preclinical	N/A	Not reported	Chen et al. 2021 [40]
JAK/STAT Inhibition					
Ruxolitinib (JAK1/2)	2 patients	Case report	HLH may be controlled	Oher aspects of the condition are progressing	Trantham et al. 2020 [128]
GM-CSF Inhibition					
Lenzilumab (anti-GM-CSF)	/	Preclinical	N/A	Not reported	Sterner et al. 2019 [58]
IFN-γ Inhibition					
Emapalumab (anti-IFN-γ)	14 patients (4 men and 10 women)	Phase II (NCT02069899, NCT03311854)	92% HLH remission	No adverse events	De Benedetti et al. 2023 [152]
Therapeutic plasma exchange	17 patients (11 men and 6 women)	/	82.3% CRS remission	No adverse events	Pu et al. 2024 [139]

The CRS and ICANS grading standards in this table are based on the ASTCT guidelines. G3 represents Grade 3, G4 represents Grade 4. N/A indicates that the data is unavailable. ‘/‘ indicates that the original text did not provide specific data, and preclinical research data needs further clinical validation. The table data is sourced from relevant clinical studies and case reports, please refer to the references for details. The current research has certain limitations: most trials have small sample sizes and lack long-term follow-up data, and there is significant heterogeneity in CAR-T products between studies, which still needs further research and improvement.

Our understanding of CRS has probably evolved beyond the classical IL-6-centric model to encompass a broader cytokine network involving IL-1, IFN-γ, GM-CSF, and pyroptosis-mediated inflammation. Notably, the role of catecholamines in amplifying cytokine storms via adrenergic receptor signaling presents a novel therapeutic target, which may be demonstrated by preclinical studies with metoprolol and metyrosine. Similarly, the identification of endothelial activation and blood-brain barrier disruption as central to ICANS pathogenesis has shifted focus toward biomarkers like Ang-2 and therapeutic strategies targeting vascular integrity. For HLH, the dominance of IFN-γ and defective NK/CTL cytotoxicity may underscore the need for targeted blockade (e.g., emapalumab) alongside conventional immunosuppression.

The management of CAR-T toxicity is gradually shifting from a passive approach to an active approach. Tocilizumab remains the cornerstone for CRS, but its limitations in ICANS (due to poor CNS penetration) necessitate alternatives such as siltuximab or intrathecal corticosteroids. The success of IL-1 blockades (anakinra) in steroid-refractory cases and HLH highlights the importance of multimodal cytokine targeting. Emerging therapies like GM-CSF neutralization (lenzilumab) and JAK inhibitors (ruxolitinib) show promise in preclinical models, though clinical validation is ongoing. Notably, the “cytokine sponge” strategy—a hydrogel-based IL-6 trap—represents a groundbreaking approach to preemptively mitigate CRS without compromising CAR-T efficacy. However, this method has not yet been applied to humans, and its feasibility remains to be investigated.

While significant progress has been made in understanding and managing CAR-T cell therapy toxicities, several critical challenges remain to be addressed. First, the development of predictive biomarkers such as ferritin for HLH and Ang-2 for ICANS could enable early risk stratification and facilitate personalized therapeutic interventions. Second, optimizing the timing and sequencing of combination cytokine blockade strategies, including IL-6 and IL-1 inhibition, may enhance clinical outcomes while maintaining antitumor efficacy. Third, innovative CAR-T cell engineering approaches incorporating safety switches (e.g., dasatinib-controlled CARs) or cytokine-neutralizing domains (e.g., IL-1Ra-secreting CARs) show promise for reducing treatment-related toxicities. Addressing these key challenges may be crucial for advancing the field and improving patient outcomes.

This review consolidates the mechanistic and therapeutic landscape of CAR-T-associated toxicities, emphasizing the interplay between cytokine dynamics, immune cell activation, and endothelial dysfunction. Although various strategies have shown high safety in clinical and preclinical studies, there are still some limitations, including the following aspects: firstly, method limitations: heterogeneity of CAR-T products in different studies and variability of toxicity grading systems. Secondly, clinical applicability: Clinical research data is relatively insufficient, and long-term follow-up data is limited. Thirdly, there is a gap in basic research: animal models may not be able to fully replicate human CRS.

Thus, research on the mechanisms and treatment strategies of CAR-T post toxicity still needs to be continuously improved and developed to enrich our understanding and control. Clinical doctors should still adjust prevention and treatment plans according to actual situations in the diagnosis and treatment process to make treatment more standardized and reasonable. The integration of novel biologics, cellular engineering, and biomarker-guided protocols may be pivotal in achieving the dual goals of efficacy and tolerability. Collaborative efforts between basic researchers and clinicians are essential to translate these advances into standardized guidelines, ultimately expanding the therapeutic potential of CAR-T therapy across diverse malignancies.

## Data Availability

No datasets were generated or analysed during the current study.
